# Predicting the Spread of SARS-CoV-2 in Italian Regions: The Calabria Case Study, February 2020–March 2022

**DOI:** 10.3390/diseases10030038

**Published:** 2022-06-30

**Authors:** Francesco Branda, Ludovico Abenavoli, Massimo Pierini, Sandra Mazzoli

**Affiliations:** 1 Department of Computer Science, Modeling, Electronics and Systems Engineering (DIMES), University of Calabria, 87036 Rende, Italy; francesco.branda@unical.it; 2 Department of Health Sciences, University Magna Graecia, 88100 Catanzaro, Italy; 3 Guglielmo Marconi University, 00193 Rome, Italy; info@epidata.it; 4 SITO WEB del Gruppo Epidemiologico, EpiData.it, 24121 Bergamo, Italy; sandra.mazzoli50@gmail.com

**Keywords:** SARIMA, time series regression models, forecasting, epidemiology, COVID-19, SARS-CoV-2, Italy, Calabria

## Abstract

Despite the stunning speed with which highly effective and safe vaccines have been developed, the emergence of new variants of SARS-CoV-2 causes high rates of (re)infection, a major impact on health care services, and a slowdown to the socio-economic system. For COVID-19, accurate and timely forecasts are therefore essential to provide the opportunity to rapidly identify risk areas affected by the pandemic, reallocate the use of health resources, design countermeasures, and increase public awareness. This paper presents the design and implementation of an approach based on autoregressive models to reliably forecast the spread of COVID-19 in Italian regions. Starting from the database of the Italian Civil Protection Department (DPC), the experimental evaluation was performed on real-world data collected from February 2020 to March 2022, focusing on Calabria, a region of Southern Italy. This evaluation shows that the proposed approach achieves a good predictive power for out-of-sample predictions within one week (R-squared > 0.9 at 1 day, R-squared > 0.7 at 7 days), although it decreases with increasing forecasted days (R-squared > 0.5 at 14 days).

## 1. Introduction

In December 2019, the new Coronavirus, SARS-CoV-2 [1], emerged and into a world population without proper specific immunization. Due to its high infectivity, the virus spread worldwide, beginning the new current pandemic [2]. During the epidemic and subsequent vaccine immunization, one of the main issues was the emergence of several Variants of Concern (VOC) [3] with consequent re-emergence of new infection cases. In particular, several notable variants of SARS-CoV-2 have emerged in recent months [4], such as B.A.2 [5], which expresses a very high infectivity rate and pathogenesis, and B.A.2.2 [6], responsible in Hong Kong for the significant increase in lethality among the unvaccinated and uninfected elderly and children.

This highlighted the importance of following the contagion evolution on a temporal scale inside the various populations, especially to define the restarting moment of new cases to prevent severe clinical cases, along with ICU admissions and deaths, to determine needed non-pharmacological intervention (NPI), to test the success or failure of containment measures in place, and to guide governments and decision makers. Moreover, it has become increasingly important to define and refine epidemiological forecasting methods in order to adequately monitor population infection cases and deaths. SARIMA (X) models are an example of methods that have already been used for prediction in epidemiology, such as malaria [7], influenza-like illness [8], dengue hemorrhagic fever [9], West Nile virus [10], scarlet fever [11], human brucellosis [12], and recently, COVID-19 [13,14].

This paper presents the design and implementation of an approach based on autoregressive models to reliably forecast the spread of COVID-19 in Italian regions. The method automatically collects daily data on the number of individuals infected with SARS-CoV-2 and performs the following operations: (i) *data pre*-*processing*, which consists of turning raw data into the required format for the purposes of analysis; (ii) *predictive modeling*, which regards the training of a model to forecast the number of infections that will happen in a specific area; (iii) *results visualization*, which presents the results in a graphical way, allowing users to visually explore the data.

As a case study, we present here the analysis of the regional trend of SARS-CoV-2 spread in Calabria, a region of Southern Italy, based on open-access coronavirus data provided by the Italian Civil Protection Department (DPC), starting from 24 February 2020. The results of the experimental evaluation show the effectiveness of the method, by achieving good accuracy in new infections forecasting within one week.

The rest of the paper is organized as follows. Section 2 outlines the problem statement and describes the proposed approach in detail. Section 3 presents the experimental evaluation. Finally, Section 4 and Section 5 conclude the paper and plan future research works.

## 2. Materials and Methods

We begin by outlining a proper notation to be used throughout the paper. Let *D* be a dataset collecting epidemiological data, where each *d*${}_{i}$ is described by the following tuple: 〈ID, T, NC, DE, RC, HOSP, ICU, TESTS〉, where *ID* is the identifier of a region, *T* is the notification date of an infection, NC indicates new positive cases, DE is the total amount of deaths, RC refers to the total number of recovered persons, HOSP and ICU are hospitalized patients with symptoms and in intensive care, respectively, and TESTS is the number of tests performed. Let $\hat{T}$ = $\langle {\hat{t}}_{1},{\hat{t}}_{2},\ldots ,{\hat{t}}_{K}\rangle$ be an ordered timestamp list and *H* = $\langle {\hat{t}}_{j},{\hat{t}}_{j\;+\;1},\ldots \rangle$ a future temporal horizon, with *j* > *K*.

As mentioned before, the main goal of this work is to reliably predict the number of new infections at a given timestamp ${\hat{t}}_{j}$ ∈ *K*. This is achieved in two steps: (*i*) estimating the COVID-19 epidemic risk in Italian regions for identifying high-risk areas (i.e., areas with particularly high risk of infection due to a high incidence of spread of SARS-CoV-2); (*ii*) training a model that given a timestamp ${\hat{t}}_{j}$ ∈ *H* states the number of new cases *N* ∈ *NC* that are predicted to happen in such areas at the timestamp ${\hat{t}}_{j}$.

**Figure 1:**
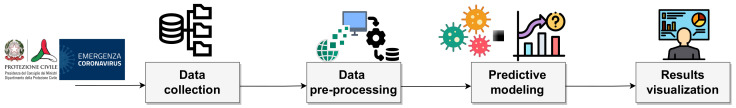
Proposed approach steps.

Figure 1 presents the general idea of the approach through a graphic representation of the whole process as a sequence of three steps. The input data of the analysis is the set of collected epidemiological data to be processed. The second step consists of cleaning, selecting, and transforming raw data into the desired format so that useful information can be derived from it. In other words, (i) incorrect and incomplete data are removed; (ii) a subset of data is selected to make it suitable for analysis; (iii) third-party data from an external authoritative source are merged with the existing database to enrich collected data. The third step is aimed at detecting the regions mostly affected by the pandemic and extracting a prediction model for providing dynamic monitoring of the spread of the disease, and supporting organizations in the evaluation of the effect of local containment measures. Finally, results visualization is performed by using an interactive dashboard to inform citizens in an intuitive way and make the collected data available.

**Figure 2:**
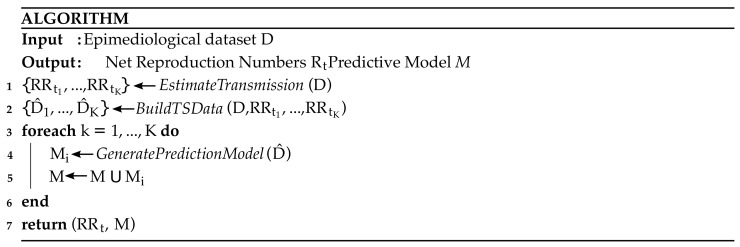
Workflow of the *predictive modeling* step.

Figure 2 reports the meta-code of the *predictive modeling* step. In particular, given a specific epidemiological dataset, the *EstimateTransmission*() method estimates the net reproduction numbers ($R_{t}$) (i.e., the average number of new cases generated by an infectious case at a given time of the epidemic) based on confirmed cases reported to the National Integrated Surveillance System (https://www.epicentro.iss.it/en/coronavirus/sars-cov-2-integrated-surveillance, accessed on 28 May 2022) and stratified by region. Methodological details can be found in [15].

As soon as this step is completed, the epidemiological dataset *D* is transformed in *K* time series datasets. Specifically, this task is executed by the *BuildTSData*(), which transforms *D* in the time series dataset collection $\hat{D}$ = {${\hat{D}}_{1},\ldots ,{\hat{D}}_{K}$} where each ${\hat{D}}_{i}$ is the time series of new cases of COVID-19 in a region $RR_{ti}$ ∈ $RR_{t}$.

Finally, for each ${\hat{D}}_{i}$, the *GeneratePredictionModel*() method generates a model *M* to forecast the number of cases that will happen in the specific region $RR_{ti}$. This task is performed using SARIMA (p,d,q)(P,D,Q)[s] model, i.e., a further development of Autoregressive Integrated Moving Average ARIMA (p,d,q) with seasonality, where *p* is the number of AR autoregression terms, *d* is the difference order, *q* is the number of MA sliding average terms, *P* refers to the maximum lag order of the seasonal autoregression term, *Q* is the maximum lag order of the moving average operator, *D* is the seasonal difference order, and *s* is the seasonal difference cycle step [16]. SARIMA (p,d,q)(P,D,Q)[s] corresponds to the polynomial operators formula

(1) $$\phi_{p}\left(B\right)\Phi_{P}\left(B^{s}\right){\left(1-B\right)}^{d}{\left(1-B^{s}\right)}^{D}x_{t}\;=\;\theta_{q}\left(B\right)\Theta_{Q}\left(B^{s}\right)\varepsilon_{t}$$

where *B* is the backshift operator (or lag operator) and $B^{n}x_{t}\;=\;x_{t-n}$; $\phi_{p}\left(B\right)$ and $\Phi_{P}\left(B^{s}\right)$ are the polynomial operators of AR autoregression (the latter refers to seasonality), $\theta_{q}\left(B\right)$ and $\Theta_{Q}\left(B^{s}\right)$ are the polynomial operators of MA sliding average (the latter refers to seasonality), where a generic polynomial operator $\Omega_{m}\left(B^{n}\right)$ yields

(2) $$\Omega_{m}\left(B^{n}\right)\;=\;1-\sum_{i\;=\;1}^{m}\Omega_{i}B^{in}$$

**Figure 3:**
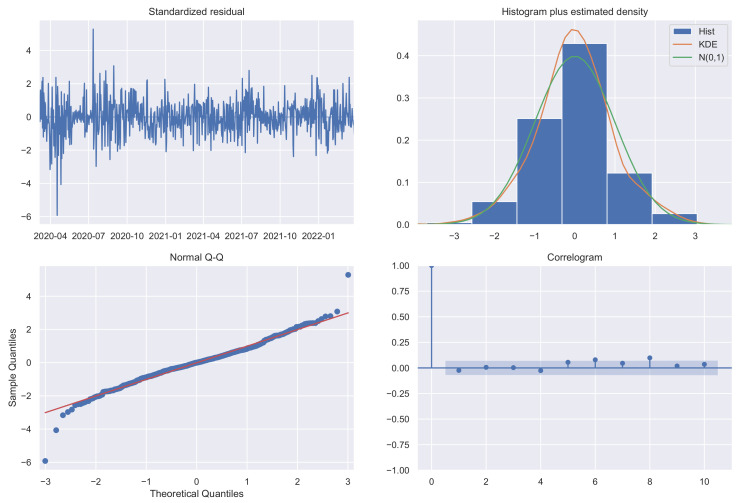
SARIMA model diagnostics.

The best model, balancing between complexity and adjustment, has been chosen via a grid-search to optimize the Bayesian Information Criterion (BIC) [16] resulting in a SARIMA (0, 1, 1)(1, 0, 1)(7) corresponding to the polynomial operators formula

(3) $$\begin{array}{c} \;\;\;\phi_{0}\left(B\right)\Phi_{1}\left(B^{7}\right){\left(1-B\right)}^{1}{\left(1-B^{7}\right)}^{0}x_{t}\;=\;\theta_{1}\left(B\right)\Theta_{1}\left(B^{1}\right)\varepsilon_{t} \end{array}$$

(4) $$\begin{array}{c} \;\;\;\;\;\;\;\;\;\;\Phi_{1}\left(B^{7}\right)\left(1-B\right)x_{t}\;\;=\;\;\theta_{1}\left(B\right)\Theta_{1}\left(B^{7}\right)\varepsilon_{t} \end{array}$$

(5) $$\begin{array}{c} \;\;\;\;\;\;\left(1-\Phi_{1}B^{7}\right)\left(1-B\right)x_{t}\;=\;\left(1-\theta_{1}B\right)\left(1-\Theta_{1}B^{7}\right)\varepsilon_{t} \end{array}$$

(6) $$\begin{array}{c} \;\left(1-B-\Phi_{1}B^{7}\;+\;\Phi_{1}B^{8}\right)x_{t}\;=\;\left(1-\Theta_{1}B^{7}-\theta_{1}B\;+\;\Theta_{1}\theta_{1}B^{8}\right)\varepsilon_{t} \end{array}$$

(7) $$\begin{array}{c} x_{t}-x_{t-1}-\Phi_{1}x_{t-7}\;+\;\Phi_{1}x_{t-8}\;=\;\epsilon_{t}-\Theta_{1}\varepsilon_{t-7}-\theta_{1}\varepsilon_{t-1}\;+\;\Theta_{1}\theta_{1}\varepsilon_{t-8} \end{array}$$

which yields the explicit formula

(8) $$x_{t}\;=\;x_{t-1}\;+\;\Phi_{1}\left(x_{t-7}-x_{t-8}\right)\;+\;\varepsilon_{t}-\theta_{1}\varepsilon_{t-1}-\Theta_{1}\left(\varepsilon_{t-7}-\varepsilon_{t-8}\right)$$

where $x_{t}$ is the observed variable at time *t*, $\varepsilon_{t}$ is the white noise, identically and independently normally distributed with mean 0 and variance $\sigma^{2}$, and $\Phi_{1}$, $\theta_{1}$, and $\Theta_{1}$ are the parameters to be estimated. The chosen model shows fairly acceptable diagnostics (standardized residuals, estimated density, Q–Q plot, and correlogram) [17,18], as can be seen in Figure 3.

### Software

All analyses were performed in Python 3.8.3 with: *statsmodels* (https://www.statsmodels.org/stable/index.html, accessed on 28 May 2022), *pmdarima* (https://pypi.org/project/pmdarima/, accessed on 28 May 2022) and *scipy* (https://scipy.org/, accessed on 28 May 2022). Plots have been created with *matplotlib* (https://matplotlib.org/, accessed on 28 May 2022) and *seaborn* (https://seaborn.pydata.org/, accessed on 28 May 2022). The complete Jupyter notebook Python code is publicly available on GitHub at https://github.com/maxdevblock/covid19_sarima_calabria, accessed on 28 May 2022.

## 3. Results

The data that we used to evaluate the effectiveness and accuracy of the approach described above are gathered from the Italian Civil Protection Department (DPC) [19] dataset and enriched at the provincial level by using an automatic scraper that extracts and transforms data from the website of the Calabria region (https://regione.calabria.it/website/, accessed on 28 May 2022) into a machine-readable format in order to make its reuse easier [20] (text in Italian). The database is freely accessible at https://github.com/fbranda/covid19-opendata-calabria, accessed on 28 May 2022. Moreover, data are graphically consultable on the COVIDA platform at https://covida.ml/, accessed on 28 May 2022.

We carried out an experimental evaluation by analyzing the SARS-CoV-2 trend in the period between 24 February 2020 and 27 March 2022, in Calabria, one of the Italian regions, which has been most affected during the fourth wave of the pandemic.

**Figure 4:**
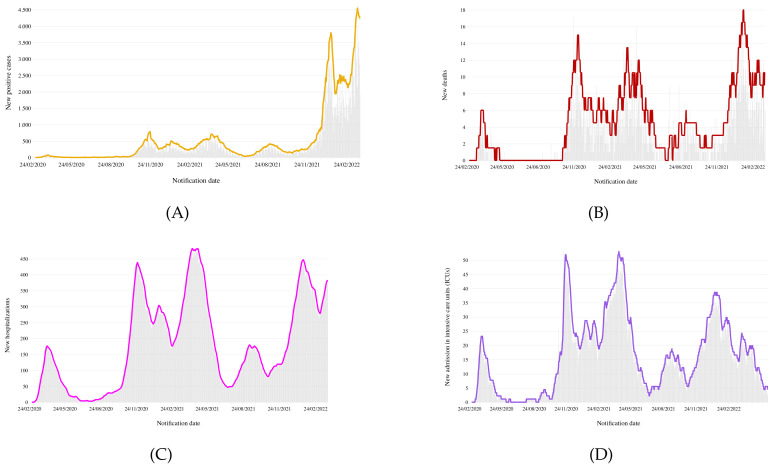
Calabria epidemiological data: (**A**) new positive cases; (**B**) total amount of deaths; (**C**) hospitalized patients with symptoms and (**D**) in intensive care.

Figure 4 shows a preliminary view of the collected epidemiological data. Specifically, Figure 4A reports the time plot of the new confirmed cases, in which new positive cases are plotted versus the time of notification. From the plot, we see that the number of cases exhibits a stable trend until December 2021, followed by a sharp increase in infections in January 2022, due to the circulation of the SARS-CoV-2 Omicron variant.

Omicron’s higher infection rate has pushed health systems to the breaking point, causing a significant number of deaths and hospitalizations. A clearer view of the Omicron’s wave can be seen in Figure 4B,C, which show the number of confirmed COVID-19 deaths and hospitalizations per day, respectively. In particular, the new record for deaths in one day was 18 on 28 January 2022 (previously it was 17 on 23 November 2020), whereas the hospitalizations have risen significantly, approaching the values of the prior wave (446 hospitalizations on 18 January 2022 versus 482 hospitalizations on 26 April 2021).

However, for the innate features of Omicron and the protection offered by the vaccines, a smaller percentage of COVID patients were admitted to intensive care units (ICUs), as shown in Figure 4D. The chart clearly shows that during the first phase of Omicron surge, an initial increase in patients (with 38 persons admitted to ICU on 11 January 2022) was followed by a smooth decreasing trend.

**Figure 5:**
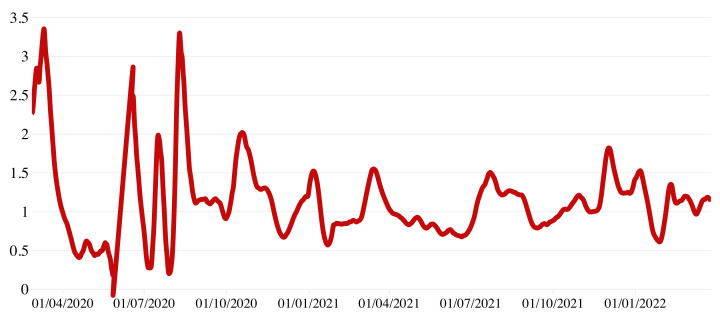
COVID-19 estimated $R_{t}$ in Calabria over a 7-day moving average, 24 February 2020–27 March 2022.

To evaluate the impact of SARS-CoV-2 circulation, we analyzed the net reproduction number $R_{t}$ (i.e., the transmission potential at a given time *t* of the epidemic once interventions are introduced or the susceptibility in the population decreases), calculated by the research group CovidStat INFN (https://covid19.infn.it/progetto.html, accessed on 28 May 2022). This method uses the growth rate determined over the last 14 days with an exponential fit to the number of infected persons per day [21] assuming the mean value of the generation time published by Cereda et al. [22]. As shown in Figure 5, with the national lockdown imposed in early March 2020, $R_{t}$ estimates followed a constantly decreasing trend. Since late May 2020 with the gradual reopening of all activities, $R_{t}$ started to fluctuate, reaching maximum values around 3 in the week from 3 to 10 August. From 7 January 2021 to 27 March 2022, $R_{t}$ remained nearly constant at values around 1.5–1.8.

**Figure 6:**
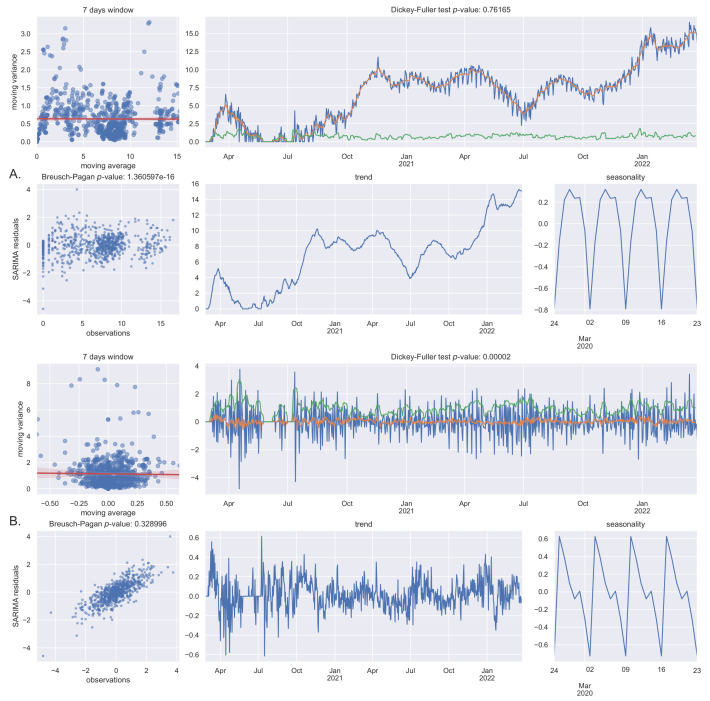
Data tests: (**A**) Box–Cox transformed data; (**B**) Box–Cox transformed and differentiated data.

To forecast new COVID-19 cases for the next 14 days, a seasonal autoregressive integrated moving average without exogenous variables (SARIMA) model was used. The data are characterized by heteroskedasticity (Breusch–Pagan p≪0.01), non-stationarity (Dickey–Fuller p = 0.76), and seasonality (7 day cycles), as shown in Figure 6.

We call “seasonality” the 7-days (circaseptan) observed oscillations because it has been treated as a seasonal behavior in the SARIMA model, even if, usually in epidemiology, the seasonality refers to longer periods (such as months). In some countries, additional hemicircaseptan (3.5 days) and 14-days periodicities have been observed [23]. The circaseptan seasonality of COVID-19 new daily cases, which has not been observed or reported in prior epidemics, is believed to be likely associated with epidemiological and social factors, mainly testing bias and reporting bias [23,24,25].

Since, as stated above, such a circaseptan seasonality has not been observed previously in prior epidemics, this study cannot be compared to prior studies but similar SARIMA models have been recently tested using circaseptan seasonality to forecast 14 or 28 days [13,14,26] with comparable results.

We have chosen not to solve heteroskedasticity but to treat it as an inherent feature also because a transformation can damage results interpretability and not completely solve the issue [27] that could be reduced with differentiation only. Nevertheless, the data have been transformed with the Box–Cox method in order to avoid negative results [28].

**Figure 7:**
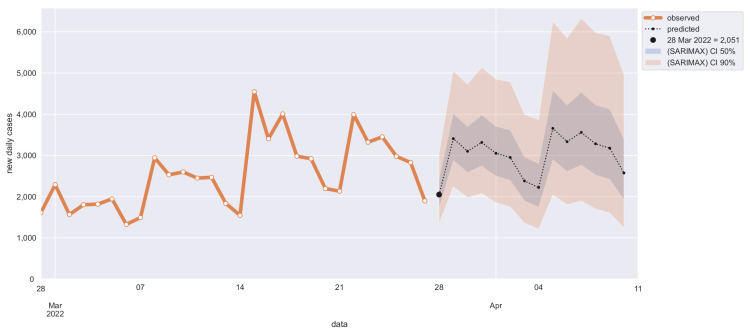
Out-of-sample 14-days prediction of daily new COVID-19 cases in Calabria with SARIMA model.

Non-stationarity can be solved by first-order differentiation, i.e., SARIMA parameter d = 1 (Dickey–Fuller p≪.01). As expected, differentiating can partially solve heteroskedasticity too (Breusch–Pagan p = 0.33). The 7-days cycle seasonality has been confirmed with both seasonal decomposition and periodogram [29] that clearly shows first and second harmonic (7 and 3.5 days). Confidence intervals of 50% and 90% have been chosen for out-of-sample 14 days’ new cases predictions (see Figure 7).

**Figure 8:**
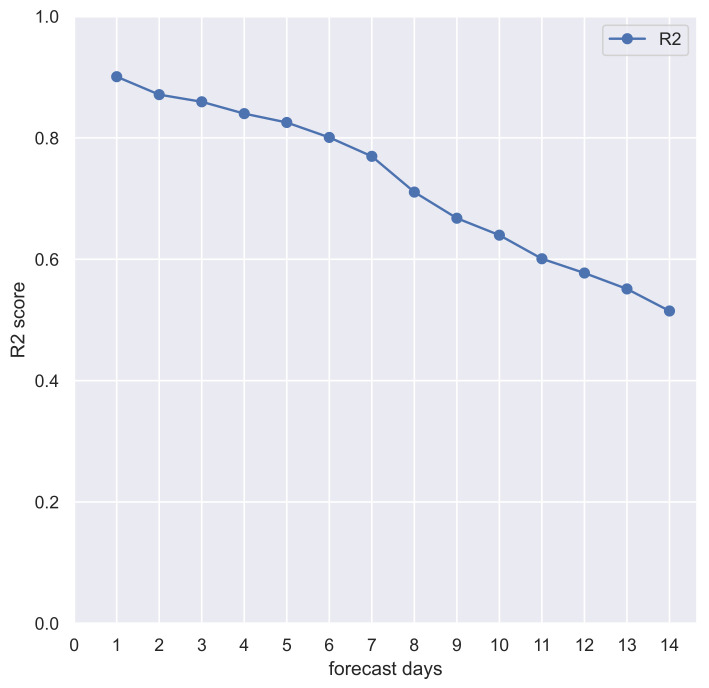
Pooled $R^{2}$ scores of the 14-days out-of-sample forecast of COVID-19 new daily cases in Calabria (Italy) with SARIMA model.

To define the SARIMA model R-squared ($R^{2}$) score for out-of-sample 14-days prediction, for each day between 1 January 2021, and 13 March 2022, we have chosen the best SARIMA model via grid-search (based on previous observations) and forecasted the next 14 days. Pooled $R^{2}$ score of 14 days of observations and predictions is 0.90 at 1 day, greater than 0.80 within 6 days, and greater than 0.50 up to 14 days (see Figure 8 and Table 1). These results confirm the appropriateness of the autoregressive model and its good performance in the epidemiology domain over rolling time horizons.

## 4. Discussion

Italy was the second country to have a large outbreak of infections of novel severe acute respiratory syndrome coronavirus 2 (SARS-CoV-2), with clusters of cases detected in Lombardy and Veneto on 21 February 2020, and the first deaths on 22 February 2020 [30,31]. By the beginning of March, the virus had spread to all regions of Italy, and to reduce the burden of the epidemic on the healthcare settings, the government imposed a national lockdown [32]. On 27 December 2020, Italy launched the vaccination campaign, which has significantly reduced the risk of COVID-19 diagnosis and COVID-19-related hospitalization and death, particularly starting 14 days from receipt of the second dose; however, the emergence of new SARS CoV-2 variants underscores the importance of receiving a third dose of COVID-19 vaccine to protect the high-risk populations (i.e., older adults) [33].

Despite the rapid development of safe and effective vaccines, there is a need to reduce viral replication, especially if new variants are associated with higher rates of (re)infection or more severe disease. An ever-increasing volume of epidemiological data offers the opportunity to apply data analytics methodologies to extract useful models able to automatically detect both which areas of the country have the highest diagnoses and how the transmission rate of each specific area varies with respect to the time period. This knowledge allows us to dynamically monitor the spread of the disease, offering the opportunity to make better policies to overcome the problem.

Overall, there are three main types of statistical modeling used for predicting infectious disease spread [34]. The first one among them is the *distribution fitting* technique, wherein most of the infectious diseases as a large number of cases are infected is fitted to the observed data and the parameters of the distribution are estimated based on the sample observations. For example, Hamzaha et al. [35] analyzed worldwide COVID-19 data to predict new cases, deaths, and recoveries using distribution methods.

A second type of infectious disease modeling includes *epidemiological models* (e.g., SIR and SEIR), which aim to describe, analyze, and understand the patterns of infectious disease. During the COVID-19 pandemic, several works investigated the effects of non-pharmaceutical measures (such as school closures, travel bans, and national lockdowns) on the spread of COVID-19. Specifically, extensions of well-established Susceptible–Infectious–Recovered (SIR) and Susceptible–Exposed–Infectious–Recovered (SEIR) models have been proposed to model the spread of COVID-19 [36,37,38]. Such analyses are typically based on complex compartmental models, which focus on individual-level dynamics.

This is a rather different approach as compared to our method, which uses the *time series modeling* technique, as detailed described in Section 2. In particular, we defined a general methodology to estimate and visualize differences in the spread of the pandemic at the national level, with a predefined (but extensible) set of steps (i.e., data collection, pre-processing, analysis, and visualization). In this way, data scientists and analysts can efficiently design and execute their applications dealing with epidemiological data. In fact, an important advantage of the work is that the users can download the Python code to reproduce the results presented in this paper and monitor the evolution of the pandemic. Moreover, the code could be adapted without much work to monitor the COVID-19 pandemic in other regions, or for future outbreaks of other infectious diseases.

The potential limitations of our study are the following: (*i*) the data provided by the Department of Civil Protection refer to the number of reported cases, which underestimates the real number of positive cases in the population; (*ii*) the data do not allow us to ascertain the date of onset of the infection, so the model will suffer from a delay relative to the trend of the infections in the population; (*iii*) the SARIMA model used, contrary to SARIMAX, does not take into account exogenous variables that could have effects both on the trend and seasonality; (*iv*) our model is limited to short-term prediction because, with current data, the only observable seasonality has a period of 7 days; (*v*) this model does not take into account the effect of case spikes due to sudden mutations of the virus and further research is needed to explore such effects on SARIMA model predictions.

Similar SARIMA models can be used for short-term predictions also in other regions and/or countries [13,14,26] but the best model, optimizing Bayesian Information Criterion (BIC) or Akaike Information Criterion (AIC), should be chosen based on available data. A similar model is currently used on the website epidata.it to forecast the next 14 days of new daily COVID-19 cases in Italy https://www.epidata.it/Italia/ARIMA.html, accessed on 28 May 2022.

Thus, this study confirms that SARIMA models can be used for short-term predictions (14 days) considering the circaseptan oscillations as seasonality, even at regional level. The best model needs to be chosen via a grid-search to optimize BIC or AIC based on available data.

## 5. Conclusions

Experimental evaluation, focusing on Calabria, showed a good predictive power for out-of-sample predictions within one week ($R^{2}$ > 0.7), whereas the predictions up to 14 days should be treated with caution since the predictive power decreases with increasing out-of-sample forecast periods. In future work, other research issues may be investigated. First, we may identify potential exogenous variables to define a better performing SARIMAX model on medium-to-long-term predictions. Second, we will extend the use of the model at the province level, for quickly identifying the potential risk areas of a region, as well as to explore the use of these models to predict the trend of other kinds of events (e.g., deaths and hospitalizations).

## Figures and Tables

**Table 1:** Pooled $R^{2}$ score for each forecasted day.

Forecasted Days	$R^{2}$
1	0.90
2	0.87
3	0.86
4	0.84
5	0.82
6	0.80
7	0.77
8	0.71
9	0.67
10	0.64
11	0.60
12	0.58
13	0.55
14	0.51

## Data Availability

Not applicable.
